# Empathy, Moral Sensitivity, and Prosocial Behavior Among Medical Undergraduates in a South Indian Tertiary Care Teaching Institute: An Analytical Cross-Sectional Study

**DOI:** 10.7759/cureus.70392

**Published:** 2024-09-28

**Authors:** Sagnika Chowdhury, Naveen K G, Robin T Vavachan

**Affiliations:** 1 Department of Community Medicine, Saveetha Medical College and Hospital, Saveetha Institute of Medical and Technical Sciences, Saveetha University, Chennai, IND

**Keywords:** empathy, ethical decision-making, gender differences, medical education, medical students, moral sensitivity, prosocialness

## Abstract

Introduction: Empathy, moral sensitivity, and prosocialness are critical traits for medical professionals, as they affect patient care quality and ethical decision-making. These traits help address psychological complexities like stress and burnout in medical practice. Despite their importance, the relationships between these traits, especially among medical students, remain underexplored. This study aims to assess empathy, moral sensitivity, and prosocialness among medical students in South India, focusing on gender differences and their associations.

Materials and methods: This cross-sectional study was conducted at Saveetha Medical College, Saveetha Institute of Medical and Technical Sciences, Chennai, with 369 medical undergraduates. Data were collected using three standardized tools: the Interpersonal Reactivity Index for empathy, the Moral Sensitivity Questionnaire, and the Prosocialness Scale for Adults. The questionnaire, including demographic data, was administered online with anonymity and consent. Descriptive statistics summarized demographic data. Inferential analyses, including t-tests, chi-square tests, Pearson correlations, and multivariate regression, examined relationships between empathy, moral sensitivity, pro-social behavior, and demographics. Statistical significance was set at p < 0.05. Ethical approval was obtained from Saveetha Medical College’s Ethics Committee.

Results: The mean age of participants was 20.5 ± 2 years, with 183 males (49.6%) and 186 females (50.4%). The majority were first-year students (29.8%), followed by second-year (19.5%) and final-year part I students (19.2%). Final professional part II students and interns comprised 15.4% and 16%, respectively.

Females scored significantly higher in empathy domains like perspective-taking (15 ± 3.3 vs. 14 ± 3.4), fantasy (14.4 ± 4 vs. 13.6 ± 3.3), and empathetic concern (15.3 ± 3.5 vs. 14.2 ± 3.1), with p values <0.05. Overall, 186 females (50.4%) had a mean empathy score of 59 ± 9, compared to 183 males (49.6%) with 56.2 ± 7.1 (Cohen's d = 0.33).

In moral sensitivity, females scored higher in modifying autonomy (7 ± 2.3 vs. 6.3 ± 2.2), structuring moral meaning (4.6 ± 2 vs. 4.15 ± 1.6), and expressing benevolence (16 ± 4 vs. 15 ± 4), with significant differences (p < 0.05). The total moral sensitivity score was higher in females (57 ± 10 vs. 54 ± 9), with a moderate effect size (Cohen's d = 0.3). Males scored higher in prosocialness, with 183 males (49.6%) scoring 54 ± 7, compared to 186 females (50.4%) with 51.5 ± 7.2 (Cohen's d = 0.3).

Weak but significant correlations were found between empathy and prosocialness (r = 0.132, p = 0.011) and between moral sensitivity and prosocialness (r = 0.479, p < 0.001). Regression analysis identified gender, prosocial behavior, and specific moral sensitivity dimensions as significant predictors of empathy scores.

Conclusion: This study revealed significant gender differences in empathy, moral sensitivity, and prosocialness among medical students. Females scored higher in empathy and moral sensitivity, whereas males showed greater prosocial behavior. The positive correlations between empathy and prosocialness and between moral sensitivity and prosocialness highlight their interconnectedness. Educational interventions that target empathy and moral sensitivity could help nurture more compassionate, ethical healthcare professionals. Future research should explore how these traits evolve throughout medical education.

## Introduction

Empathy, moral sensitivity, and prosocialness are fundamental attributes in the medical profession, deeply influencing the quality of care provided to patients and the ethical decision-making processes of healthcare professionals. Physicians often face heavy workloads, long working hours, and high-pressure environments, all of which can lead to psychological challenges such as anxiety, depression, and burnout. These challenges, in turn, can negatively affect the well-being of physicians and the care they provide [1,2].

Fostering strong patient connections and enhancing treatment success require empathy, which is defined as the capacity to comprehend and experience another person's feelings. Empathy involves a complex interplay between cognitive and emotional processes, requiring physicians to recognize and temporarily experience their patients' emotional states while effectively communicating this understanding [3]. Cultivating empathy during undergraduate medical education is thus critical, shaping future physicians' abilities to deliver compassionate care [4].

Equally important is moral sensitivity, a vital professional competency that enables physicians to navigate the ethical complexities of patient care. Moral sensitivity involves recognizing and accurately diagnosing moral issues, which guides ethical decision-making and enhances moral behavior in clinical practice. It encompasses several dimensions, including moral responsibility, strength, and burden, all contributing to a physician's ability to make decisions that prioritize patient welfare [5,6].

Prosocialness refers to voluntary behaviors aimed at benefiting others, such as helping, caring for, assisting, or comforting them [7]. The word altruism has sometimes been used to describe a specific subset of prosocial behaviors, such as self-sacrifice or helping without expecting external rewards. However, this usage can be misleading because altruism is fundamentally a motivational concept. Altruism refers to the motivation behind the behavior, specifically the desire to improve another person's welfare rather than the behavior itself. Therefore, while altruistic actions may result in helping others, the key distinction is that altruism focuses on the intention behind the action rather than the act itself [8]. To date, medical education has primarily emphasized altruism rather than prosocialness [9]. Moreover, moral sensitivity is closely related to prosocial behavior and empathy [10,11].

Although the significance of empathy, moral sensitivity, and prosocialness is well-established, the relationships between these qualities remain underexplored, particularly in medical education. This research delves into gender differences in these attributes among medical students and examines how they are interconnected. The findings are intended to offer insights that could guide the creation of educational approaches to nurturing these essential traits, ultimately shaping compassionate and ethically responsible healthcare professionals.

## Materials and methods

This study, employing an analytical cross-sectional study, was conducted at Saveetha Medical College and Hospital, Saveetha Institute of Medical and Technical Sciences (SIMATS), Chennai, Tamil Nadu, in the month of January 2024. The study aimed to assess empathy, moral sensitivity, and prosocialness among the students, as well as the correlation between these attributes. Data were collected using three standardized tools: the Interpersonal Reactivity Index (IRI) [12], the Moral Sensitivity Questionnaire (MSQ) [13], and the Prosocialness Scale for Adults (PSA) [7]. A structured questionnaire incorporating these tools, along with basic details such as academic year, gender, and age, was administered to the participants.

The IRI [12] was developed to evaluate empathy and consists of 28 questions with a 5-point Likert scale (ranging from "does not describe me well" to "describes me very well"). It includes four subdomains, seven questions each: fantasy (resonating with fictional characters), perspective-taking (seeing things from others' perspectives), empathetic concern (displaying care, warmth, and concern for others), and personal distress (feeling troubled and uneasy due to another's unpleasant experiences). Each domain's score ranges from 0 to 28, with the total score representing the sum of these four areas, where higher scores reflect greater empathy [12,14,15].

The MSQ was developed by Lützén et al. [13]. Initially comprising 30 questions, the MSQ was later revised to 25 items [16]. It assessed nurses' moral judgment during clinical care across six domains: modifying autonomy and experiencing moral conflict (each with three items), interpersonal orientation and reliance on medical authority (each with five items), structuring moral meaning (two items), and expressing benevolence (seven items). Items were rated on a scale from 0 to 4, where 0 represents "completely disagree" and 4 represents "completely agree." The total score could range from 0 to 100, with scores from 0 to 50 indicating low moral sensitivity, 51 to 75 representing moderate sensitivity, and 76 to 100 reflecting high moral sensitivity. The reliability of the MSQ has been proven in many parts of the world, making it a robust tool for assessing moral sensitivity across different contexts [17,18].

The PSA, developed by Caprara et al. [7], includes 16 items and is rated on a scale from 1 (never) to 5 (always). Higher scores imply a greater degree of prosocial activity. The questionnaire (see Appendix 1) was incorporated into a Google Form (Google, Mountain View, CA), including a consent page (see Appendix 2) at the beginning to ensure informed participation. Only participants who provided consent were granted access to the rest of the questionnaire. If consent was not provided, they were excluded from the study, and the form automatically ended without allowing access to the full questionnaire. This approach ensured informed participation and maintained the ethical standards required for the study. The form was circulated among medical undergraduates (including interns) through their WhatsApp groups, QR codes posted on notice boards, and via their class representatives. Participants were given one week (from January 8 to 14, 2024) to complete the form, after which it was closed for responses. This approach ensured participant anonymity and facilitated efficient data collection. A total of 369 students provided consent and submitted their entries, while 48 rejected participation. The remaining students did not attempt to open the form. Following data collection, empathy scores below the overall sample mean were classified as low, and scores at or above the sample mean were considered high. Similarly, prosocialness scores were categorized as low or high based on the mean score.

All statistical analyses were carried out with Jamovi Solid version 2.3.28 (computer software; retrieved from https://www.jamovi.org/downloads/jamovi-2.3.28.0-win64.exe). Descriptive statistics were used to summarize demographic data, while inferential analyses, including t-tests, chi-square tests, Pearson correlations, and multivariate linear regression, were applied to examine the relationships among empathy, moral sensitivity, prosocial behavior, and demographic variables. Welch's t-test was used when the assumption of equal variances was unmet. Effect sizes were determined using Cohen's d, with statistical significance set at p < 0.05. Cohen's d value of 0.2 indicates a small effect size, 0.5 suggests a medium effect, and 0.8 or greater reflects a large effect. For regression analysis, assumptions such as multicollinearity and autocorrelation were tested to validate the model.

Ethical approval for the study was obtained from the Institutional Review Board and the Institutional Ethics Committee of Saveetha Medical College and Hospital, SIMATS, Chennai, Tamil Nadu, with reference number 270/07/2024/UG/SRB/SMCH. Informed consent was obtained from all participants before they were enrolled in the study, and strict measures were followed to ensure the privacy and confidentiality of participants throughout the research.

## Results

This study involved 369 medical undergraduates from Saveetha Medical College and Hospital, SIMATS, in Chennai, Tamil Nadu. The participant's mean age was 20.5 years with a standard deviation of two years. The age ranged from 18 to 28 years. The gender distribution was as follows: males comprised 183 (49.6%) of the participants, while females accounted for 186 (50.4%).

**Figure 1:**
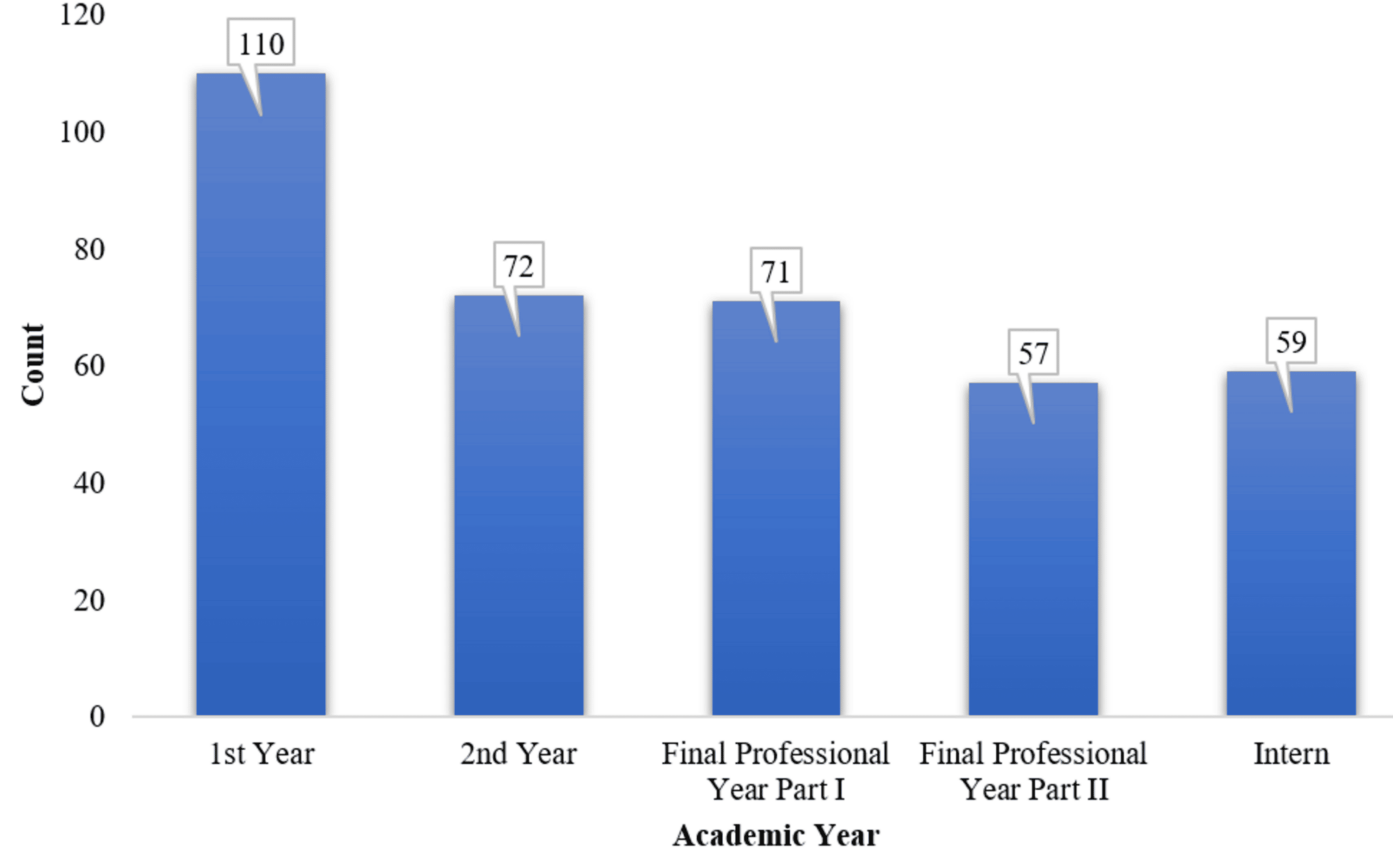
Academic year distribution of participants (N = 369)

The majority of the participants were first-year students, 110 (29.8%), followed by second-year students, 72 (19.5%), and the final professional part I students, 71 (19.2%). Final professional part II students and interns had similar representation, with 57 (15.4%) and 59 (16%), respectively (Figure 1).

In Table 1, the empathy scores across genders show that female students scored higher than males in the domains of perspective-taking (15 ± 3.3 vs. 14 ± 3.4), fantasy (14.4 ± 4 vs. 13.6 ± 3.3), and empathetic concern (15.3 ± 3.5 vs. 14.2 ± 3.1). These differences were statistically significant, with p values <0.05. The overall empathy score was also higher for females (59 ± 9) compared to males (56.2 ± 7.1), with a significant p value of 0.002. The effect sizes, indicated by Cohen's d, were small to moderate (ranging from 0.22 to 0.33), suggesting that while the differences are statistically significant, the practical impact of gender on these empathy domains is modest. However, there was no significant difference in the personal distress scores between genders (p = 0.807). Due to the violation of the homogeneity of variance assumption, the Welch t statistic was used for the fantasy and overall empathy scores, where Levene's test indicated significant p values of 0.015 and 0.003, respectively.

**Table 1:** Empathy scores across gender (N = 369) *Welch t value SD: standard deviation; df: degree of freedom; NA: not applicable

Empathy domains	Male, mean (SD)	Female, mean (SD)	Overall, mean (SD)	t statistic (df)	p value	Cohen’s d
Perspective-taking	14 (3.4)	15 (3.3)	14.5 (3.4)	2.2 (367)	0.03	0.23
Fantasy	13.6 (3.3)	14.4 (4)	14 (4)	2.1 (348)^*^	0.03	0.22
Empathetic concern	14.2 (3.1)	15.3 (3.5)	15 (3.3)	3 (367)	0.003	0.31
Personal distress	14.3 (3.3)	14.4 (3.1)	14.3 (3.2)	0.24 (367)	0.807	NA
Empathy overall	56.2 (7.1)	59 (9)	57.6 (8.3)	3.2 (346)^*^	0.002	0.33

Female students scored higher than males in the domains of Modifying Autonomy, Structuring Moral Meaning, and Expressing Benevolence, with statistically significant differences (p = 0.04, 0.011, and 0.015) and small effect sizes (Cohen's d = 0.214-0.28) (Table 2). The overall Moral Sensitivity score was also higher for females (57 ± 10 vs. 54 ± 9) with a moderate effect size (Cohen's d = 0.3). No significant gender differences were observed in Interpersonal Orientation, Reliance on Medical Authority, and Experiencing Moral Conflict (p = 0.19, 0.215, and 0.631) (Table 2).

**Table 2:** Moral sensitivity scores across gender (N = 369) SD: standard deviation; df: degree of freedom; NA: not applicable

Moral sensitivity domains	Male, mean (SD)	Female, mean (SD)	Overall, mean (SD)	t statistic (df)	p value	Cohen’s d
Modifying autonomy	6.3 (2.2)	7 (2.3)	6.6 (2.2)	2 (367)	0.04	0.214
Interpersonal orientation	11.5 (3)	12 (3)	12 (3)	1.3 (367)	0.19	NA
Structuring moral meaning	4.15 (1.6)	4.6 (2)	4.4 (1.7)	2.5 (367)	0.011	0.28
Reliance on medical authority	11 (3)	11.2 (2.7)	11 (3)	1.2 (367)	0.215	NA
Experiencing moral conflict	6.4 (2)	6.5 (2)	6.5 (2)	0.5 (367)	0.631	NA
Expressing benevolence	15 (4)	16 (4)	15.3 (4)	2.4 (367)	0.015	0.25
Moral sensitivity overall	54 (9)	57 (10)	55.4 (9.3)	3 (367)	0.004	0.3

Male students outscored their female counterparts in terms of prosocialness, with male students having a mean score of 54 ± 7 compared to female students' mean score of 51.5 ± 7.2. Cohen's d (0.3) indicated that this difference had a moderate effect size and was statistically significant (p = 0.002). Due to unequal variances, the Welch t statistic was used for this analysis (Table 3).

**Table 3:** Prosocialness scores across gender (N = 369) SD: standard deviation; df: degree of freedom

Attribute	Male, mean (SD)	Female, mean (SD)	Welch t statistic (df)	p value	Cohen’s d
Prosocialness	54 (7.7)	51.5 (6.6)	3 (356)	0.002	0.3

Table 4 presents the association between academic year and levels of empathy, moral sensitivity, and prosocialness. The chi-square analysis reveals a significant association between academic year and empathy levels, particularly for students in their final professional year part I, where a higher number of students scored low on empathy, χ²(4) = 10, p = 0.04. Similarly, a significant association exists between academic year and prosocialness, with a notable difference observed in final professional year part I students, χ²(4) = 10, p = 0.044. However, no significant association was found between academic year and moral sensitivity, χ²(8) = 12, p = 0.153 (Table 4).

**Table 4:** Association between academic year and levels of empathy, moral sensitivity, and prosocialness (N = 369) χ²: chi-square coefficient; df: degree of freedom

Academic year	Empathy				Moral sensitivity					Prosocialness			
	Low	High	χ^2 ^(df)	p value	Low	Moderate	High	χ^2 ^(df)	p value	Low	High	χ^2 ^(df)	p value
First year	55	55	10 (4)	0.04	34	71	2	12 (8)	0.153	75	35	10 (4)	0.044
Second year	31	41			20	49	2			47	25		
Final professional part I	35	36			18	50	2			38	33		
Final professional part II	36	21			28	29	0			28	29		
Intern	39	20			19	37	9			30	29		

There was a weak positive correlation between empathy and moral sensitivity (r = 0.082), though this was not statistically significant (p = 0.115). However, empathy showed a statistically significant, albeit weak, positive correlation with prosocialness (r = 0.132, p = 0.011), with an R² value of 0.343, indicating that 34.3% of the variation in prosocialness was explained by empathy. Similarly, moral sensitivity was moderately correlated with prosocialness (r = 0.479, p < 0.001), with an R² value of 0.229, indicating that 22.9% of the variation in prosocialness was explained by moral sensitivity. These results suggested that while higher levels of prosocial behavior were associated with higher empathy and moral sensitivity, empathy and moral sensitivity did not significantly correlate with each other (Table 5). These correlations does not imply causation unless proven by a multivariate regression analysis.

**Table 5:** Correlation between levels of empathy, moral sensitivity, and prosocialness (N = 369) r: correlation coefficient; df: degree of freedom

Variables/attributes	Statistical values	Empathy	Moral sensitivity
Empathy	Pearson's r	-	-
	df	-	-
	p value	-	-
Moral sensitivity	Pearson's r	0.082	-
	df	367	-
	p value	0.115	-
Prosocialness	Pearson's r	0.132	0.479
	df	367	367
	p value	0.011	<0.001

The regression model in Table 6 aimed to predict the total empathy scores among medical undergraduates based on various predictors such as academic year, age, sex, moral sensitivity domains, and prosocialness scores. The overall model demonstrated an adjusted R² value of 0.14, indicating that 14% of the variance in empathy scores could be explained by the predictors and a significant overall model fit, F(13, 355) = 5.5, p < 0.001. Significant predictors included gender, with female participants showing higher empathy scores (p = 0.02), and prosocialness, which positively correlated with empathy scores (p = 0.01). Additionally, expressing benevolence positively influenced empathy (p = 0.04), while interpersonal orientation and moral autonomy negatively influenced empathy scores. Assumption checks indicated no significant issues with autocorrelation (Durbin-Watson statistic = 1.82) or multicollinearity (acceptable variance inflation factor values). These findings highlight the significant role of gender, prosocial behavior, and specific moral sensitivity dimensions in predicting empathy among medical students.

**Table 6:** Multivariate analysis of factors influencing empathy among medical students (N = 369) Statistical results: R^2^ = 0.17; R^2^ adjusted = 0.14; Durbin-Watson value = 1.82; F(13, 355) = 5.5; p < 0.001 CI: confidence interval; R^2^: coefficient of determination

Variable	B	(95% CI)	t	p value
Constant	57.5	38 to 77.3	5.72	<0.01
Academic year				
Second year vs. first year	1.41	-1.13 to 4	1.1	0.276
Final professional part I vs. first year	3	-0.47 to 6.5	1.7	0.090
Final professional part II vs. first year	-2.7	-7.6 to 2.23	-1.1	0.284
Intern vs. first year	-3	-8.3 to 2.23	-1.1	0.258
Female vs. male	2	0.33 to 3.6	2.4	0.02
Age in years	-0.37	-1.3 to 0.64	-0.71	0.475
Moral sensitivity domains				
Interpersonal orientation	-0.31	-0.62 to 0.48	-2	0.05
Modifying autonomy	0.1	-0.3 to 0.5	0.5	0.62
Reliance on medical authority	-0.45	-0.75 to -0.15	-3	0.003
Expressing benevolence	0.24	0 to 0.48	2	0.04
Experiencing moral conflict	0.24	-0.16 to 0.64	1.17	0.24
Structuring moral meaning	0.04	-0.45 to 0.53	0.154	0.87
Prosocialness	0.17	0.04 to 0.3	2.65	0.01

## Discussion

This study included 369 medical undergraduates from Saveetha Medical College and identified significant gender differences in empathy, moral sensitivity, and prosocialness. Females scored higher in empathy and moral sensitivity, whereas males scored higher in prosocialness. The academic year was significantly associated with empathy and prosocialness, particularly in final professional year part I students. There is a noticeable trend of decreasing empathy scores as students advance in their academic years. A weak positive correlation was observed between empathy and prosocialness, with a moderate correlation between moral sensitivity and prosocialness. Multivariate analysis highlighted gender (female), prosocialness, and expressing benevolence as significant positive predictors of empathy, whereas interpersonal orientation and reliance on medical authority had negative impacts.

Our study reveals significant gender differences in empathy, with female students consistently scoring higher across various domains. These findings align with the work of Shashikumar et al. [19], who also reported that female medical students outscored their male counterparts in empathy throughout different semesters. Shashikumar et al. observed a noticeable decline in empathy scores by the seventh semester, especially among male students [19]. However, our study did not find such a pronounced decline, highlighting how curricular structures and educational environments can impact the trajectory of empathy development.

The consistency of these gender differences is further supported by Chatterjee et al. [20], who found that female students scored higher in empathy compared to males. Chatterjee et al. noted a decrease in empathy scores from the first to the third semester, followed by a plateau and then an increase by the seventh semester [20]. In contrast, our study identified persistent gender differences across all empathy domains without observing similar fluctuations. This suggests that while gender differences in empathy are a common finding, the overall trajectory of empathy development may vary based on specific curricular and cultural contexts.

Building on this, Shi and Du [21] and Akgün et al. [22] also found that female students scored higher than males in empathy domains such as perspective-taking and empathic concern. However, Shi and Du observed higher personal distress scores among females, a finding not replicated in our study, possibly due to cultural differences, as their research was conducted in China [21]. Akgün et al. reported significant fluctuations in empathy levels during medical training, particularly a decline in clinical empathy after the third year [22]. In contrast, our study did not observe such a decline, suggesting that the development of empathy may differ across educational settings.

Hojat et al. [23] and G C et al. [24] provide additional evidence of the persistent gender gap in empathy. Both studies utilized the Jefferson Scale of Empathy and found that female students consistently scored higher. Our study, which employed the IRI, also found similar gender differences, indicating that these patterns are robust across different measurement tools. GC et al. further noted that educational interventions, such as a medical humanities module, significantly increased empathy levels, suggesting that targeted programs could help mitigate gender disparities and enhance overall empathy among medical students [23,24].

Our study's results are further corroborated by the work of Javaeed et al. [25] and Sathaporn and Pitanupong [26], both of whom highlighted gender differences in empathy, with females generally scoring higher. While Javaeed et al. found no significant difference in overall empathy scores between genders, our study identified consistent gender-based differences across multiple empathy domains [25]. Additionally, Sathaporn and Pitanupong emphasized the importance of mental health in empathy, a factor not directly assessed in our study but one that remains crucial for future research [26].

Studies by Assing Hvidt et al. [27] and Wu et al. [28] reinforce our findings, particularly the consistent observation of higher empathy scores among female students. Assing Hvidt et al. found that empathy scores remained stable across different years of medical education [27], while Wu et al. highlighted empathy's role in reducing learning burnout [28]. This suggests that empathy not only fosters resilience among medical students but also plays a critical role in their overall well-being.

In contrast to these findings, Yeo [29] and Huang et al. [30] reported instances where male students scored higher in empathy, though these results were not consistent across all years of study. Yeo observed higher empathy scores among male students during their third year [29], while Huang et al. attributed this anomaly to distress and academic pressures faced by female students [30]. These studies highlight the complexity of empathy development, which appears to be influenced by a range of factors including cultural context, stress, and the educational environment.

We couldn't find any studies directly measuring moral sensitivity among medical students. Most of the existing research has focused on nursing students [13,31,32], nursing staff, and physicians [16,33,34]. The MSQ, which we used, was originally developed for the nursing profession. After critically assessing these studies, we identified several relevant comparisons.

The concept of moral sensitivity has been extensively explored in the context of healthcare professionals, particularly in studies by Lützén et al. [34,35]. Lützén et al. [34] emphasized the significant role of clinical context and professional responsibilities in shaping moral sensitivity, with notable gender differences where female professionals often exhibited higher moral sensitivity. This foundational work highlights how moral sensitivity evolves in response to professional practice demands, although its direct applicability to medical students, who are still in training, remains somewhat limited.

Lützén et al. [35] further developed the concept of moral sensitivity, identifying key dimensions such as moral burden, strength, and responsibility. While this study focused on established professionals, the theoretical framework it provides is relevant for understanding how moral sensitivity might begin to develop during medical education. The consistency of gender differences observed in this study aligns with our findings, where female medical students scored higher in moral sensitivity, suggesting that gender is an important factor in the ethical development of healthcare professionals.

AlMahmoud et al. [36] shifted the focus to ethics education in medical schools, highlighting the preferences and perceptions of final-year medical students. The study found that female students had a stronger preference for ethics education and believed more strongly in its importance compared to their male counterparts. This is consistent with the gender differences observed in our study, where female students exhibited higher moral sensitivity. The emphasis on ethics education in AlMahmoud et al.'s study reinforces the importance of incorporating ethics into the curriculum to foster moral sensitivity among medical students [36].

Taheri et al. [33] explored the relationship between physicians' moral sensitivity and patient satisfaction, demonstrating a positive correlation between the two. This finding is directly relevant to our research, suggesting that enhancing moral sensitivity through ethics education can lead to better patient outcomes. The study's focus on the practical implications of moral sensitivity underscores its importance in ethical decision-making and improving clinical outcomes, reinforcing the need for robust ethics training in medical education.

While the studies by Lützén et al. [34,35], AlMahmoud et al. [36], and Taheri et al. [33] provide valuable insights into the development and impact of moral sensitivity, their direct applicability to medical students must be interpreted with caution due to differences in professional experience and clinical exposure. However, it is crucial to consider the emphasis on ethics education, gender differences, and the clinical relevance of moral sensitivity in developing educational strategies to enhance moral sensitivity among medical students.

No studies have directly assessed the association between empathy, moral sensitivity, and prosocialness among medical students or professionals. Additionally, there is a lack of research focusing solely on prosocialness in this context. However, related research in the nursing field, such as the study by Suazo et al. [31], offers valuable insights. Suazo et al. explored the connections between moral sensitivity, empathy, and prosocial behavior in the context of humanized nursing care. They found that cognitive empathy mediated the relationship between moral sensitivity and prosocial behavior, suggesting that empathy enhances the ability to understand and respond to others' needs in a prosocial manner. While these findings are specific to nursing professionals, they suggest that similar dynamics could be at play in medical students, emphasizing the potential importance of empathy in fostering both moral sensitivity and prosocial behavior in healthcare settings.

Our study examined the relationships between empathy, moral sensitivity, and prosocialness among medical students. Our results revealed a significant positive correlation between empathy and prosocialness, indicating that students who scored higher in empathy were more likely to engage in prosocial behaviors. Similarly, a moderate positive correlation was found between moral sensitivity and prosocialness, suggesting that students with higher moral sensitivity are more inclined toward prosocial behavior.

Furthermore, our multivariable analysis of empathy provided additional insights. The analysis demonstrated that prosocialness was a significant predictor of empathy, indicating that students with higher levels of prosocial behavior tended to have higher empathy scores. Gender also played a significant role, with female students showing higher empathy levels. Additionally, dimensions of moral sensitivity, such as expressing benevolence and reliance on medical authority, were found to influence empathy scores, highlighting the complex interplay between these variables.

These findings align with the mediating role of empathy observed by Suazo et al. [31] and suggest that in medical education, fostering empathy may enhance moral sensitivity and encourage prosocial behavior. This underscores the importance of integrating ethics education and training that emphasizes empathy and moral sensitivity, as these attributes are interconnected and collectively contribute to the development of compassionate and ethical healthcare professionals.

While direct studies on the association between empathy, moral sensitivity, and prosocialness in medical students are lacking, insights from related research in nursing care and the findings from our study highlight the importance of these attributes in medical education. The positive associations observed in our study suggest that enhancing empathy through targeted educational interventions could have a ripple effect, promoting both moral sensitivity and prosocial behavior among future physicians.

A major strength of our study is the use of validated instruments, including the IRI for empathy, the MSQ, and the PSA. These tools facilitate a thorough evaluation of these constructs in medical students. Furthermore, our study adds valuable insights to the limited research on the connections between empathy, moral sensitivity, and prosocial behavior within medical education, offering new perspectives that could help shape future educational approaches.

There were also several limitations to this study. First, the relatively small sample size may restrict the broader applicability of the results. Second, the cross-sectional nature of the study means that causative relationships between the variables cannot be established; longitudinal research would be needed to explore how these attributes evolve and interact over time during medical education. Furthermore, the study did not account for various potential confounding factors, such as prior educational experiences, individual personality traits, and cultural influences, which might have influenced the findings. Finally, using self-reported data could introduce bias, as participants may respond in ways they perceive as socially acceptable rather than reflecting their true behaviors and attitudes.

## Conclusions

Our study reveals pronounced gender differences in empathy and moral sensitivity among medical students, with female students consistently demonstrating higher scores across various domains. The significant positive correlations between empathy, moral sensitivity, and prosocialness suggest that targeted educational interventions could play a crucial role in cultivating these essential attributes, ultimately enhancing the ethical and compassionate practices of future physicians. While our findings resonate with existing research, the study's limitations, such as a relatively small sample size and the absence of controls for potential confounders, highlight the need for further investigation. Future longitudinal studies are necessary to more deeply explore the evolution and interrelationship of these key qualities throughout medical education.

## Appendices

Appendix A

**Table 7:** Final questionnaire

Question	Answer options				
Academic year of the MBBS course	First year	Second year	Final professional part - I	Final professional part - II	Intern
Gender	Male	Female	-	-	-
Age	___ years				
I daydream and fantasize about things that might happen to me	A (Does not describe me well)	B	C	D	E (Describes me very well)
I often have tender, concerned feelings for people less fortunate than me	A (Does not describe me well)	B	C	D	E (Describes me very well)
I sometimes find it difficult to see things from the “other guy's” point of view	A (Does not describe me well)	B	C	D	E (Describes me very well)
Sometimes I don't feel very sorry for other people when they are having problems	A (Does not describe me well)	B	C	D	E (Describes me very well)
I really get involved with the feelings of the characters in a novel	A (Does not describe me well)	B	C	D	E (Describes me very well)
In emergency situations, I feel apprehensive and ill-at-ease	A (Does not describe me well)	B	C	D	E (Describes me very well)
I am usually objective when I watch a movie or play, and I don't often get completely caught up in it	A (Does not describe me well)	B	C	D	E (Describes me very well)
I try to look at everybody's side of a disagreement before I make a decision	A (Does not describe me well)	B	C	D	E (Describes me very well)
When I see someone being taken advantage of, I feel kind of protective toward them	A (Does not describe me well)	B	C	D	E (Describes me very well)
I sometimes feel helpless when I am in the middle of a very emotional situation	A (Does not describe me well)	B	C	D	E (Describes me very well)
I sometimes try to understand my friends better by imagining how things look from their perspective	A (Does not describe me well)	B	C	D	E (Describes me very well)
Becoming extremely involved in a good book or movie is somewhat rare for me	A (Does not describe me well)	B	C	D	E (Describes me very well)
When I see someone get hurt, I tend to remain calm	A (Does not describe me well)	B	C	D	E (Describes me very well)
Other people's misfortunes do not usually disturb me a great deal	A (Does not describe me well)	B	C	D	E (Describes me very well)
If I'm sure I'm right about something, I don't waste much time listening to other people's arguments	A (Does not describe me well)	B	C	D	E (Describes me very well)
After seeing a play or movie, I have felt as though I were one of the characters	A (Does not describe me well)	B	C	D	E (Describes me very well)
Being in a tense emotional situation scares me	A (Does not describe me well)	B	C	D	E (Describes me very well)
When I see someone being treated unfairly, I sometimes don't feel very much pity for them	A (Does not describe me well)	B	C	D	E (Describes me very well)
I am usually pretty effective in dealing with emergencies	A (Does not describe me well)	B	C	D	E (Describes me very well)
I am often quite touched by things that I see happen	A (Does not describe me well)	B	C	D	E (Describes me very well)
I believe that there are two sides to every question and try to look at them both	A (Does not describe me well)	B	C	D	E (Describes me very well)
I would describe myself as a pretty soft-hearted person	A (Does not describe me well)	B	C	D	E (Describes me very well)
When I watch a good movie, I can very easily put myself in the place of a leading character	A (Does not describe me well)	B	C	D	E (Describes me very well)
I tend to lose control during emergencies	A (Does not describe me well)	B	C	D	E (Describes me very well)
When I'm upset at someone, I usually try to “put myself in his shoes” for a while	A (Does not describe me well)	B	C	D	E (Describes me very well)
When I am reading an interesting story or novel, I imagine how I would feel if the events in the story were happening to me	A (Does not describe me well)	B	C	D	E (Describes me very well)
When I see someone who badly needs help in an emergency, I go to pieces	A (Does not describe me well)	B	C	D	E (Describes me very well)
Before criticizing somebody, I try to imagine how I would feel if I were in their place	A (Does not describe me well)	B	C	D	E (Describes me very well)
It is my responsibility as a doctor to have knowledge of the patient’s total situation	Strongly agree	Agree	Neutral	Disagree	Strongly disagree
My work would feel meaningless if I never saw any improvement in my patients	Strongly agree	Agree	Neutral	Disagree	Strongly disagree
It is important that I should obtain a positive response from the patient in everything I do	Strongly agree	Agree	Neutral	Disagree	Strongly disagree
When I need to make a decision against the will of a patient, I do so according to my opinion about what is good care	Strongly agree	Agree	Neutral	Disagree	Strongly disagree
If I should lose the patient’s trust, I would feel that my work would lack meaning	Strongly agree	Agree	Neutral	Disagree	Strongly disagree
When I have to make difficult decisions for the patient, it is important always to be honest with him or her	Strongly agree	Agree	Neutral	Disagree	Strongly disagree
I believe that good care includes respecting the patient’s self-choice	Strongly agree	Agree	Neutral	Disagree	Strongly disagree
If a patient does not have insight into the illness, there is little I can do for him or her	Strongly agree	Agree	Neutral	Disagree	Strongly disagree
I am often confronted by situations in which I experience conflict in how to approach the patient	Strongly agree	Agree	Neutral	Disagree	Strongly disagree
I believe that it is important to have firm principles for the care of certain patients	Strongly agree	Agree	Neutral	Disagree	Strongly disagree
I often face situations in which it is difficult to know what action is ethically right for a particular patient	Strongly agree	Agree	Neutral	Disagree	Strongly disagree
I always base my actions on medical knowledge of what is the best treatment, even if the patient protests	Strongly agree	Agree	Neutral	Disagree	Strongly disagree
I rely mostly on the nurses’ knowledge about a patient when I am unsure	Strongly agree	Agree	Neutral	Disagree	Strongly disagree
Most of all, it is the reactions of patients that show me if I have made the right decision	Strongly agree	Agree	Neutral	Disagree	Strongly disagree
I often think about my own values and norms that may influence my actions	Strongly agree	Agree	Neutral	Disagree	Strongly disagree
My own experience is more useful than theory in situations in which it is difficult to know what is ethically right	Strongly agree	Agree	Neutral	Disagree	Strongly disagree
If a patient is being treated under the Mental Health Act, I expect nursing staff to follow my orders even if the patient is noncompliant	Strongly agree	Agree	Neutral	Disagree	Strongly disagree
It is important that I should have rules to follow when a patient who is not being treated under the Mental Health Act refuses treatment	Strongly agree	Agree	Neutral	Disagree	Strongly disagree
I believe that good care includes participation of the patient, even of those with severe mental disorders	Strongly agree	Agree	Neutral	Disagree	Strongly disagree
I find it difficult to provide good care against the patient's will	Strongly agree	Agree	Neutral	Disagree	Strongly disagree
Sometimes there is good reason to threaten a patient with an injection when oral medication is refused	Strongly agree	Agree	Neutral	Disagree	Strongly disagree
In situations where it is difficult to know what is right, I consult my colleagues on what to do	Strongly agree	Agree	Neutral	Disagree	Strongly disagree
I rely mostly on my intuition when I have to make a difficult decision for a patient	Strongly agree	Agree	Neutral	Disagree	Strongly disagree
As a doctor, I must always know how each of my patients should be respectfully approached	Strongly agree	Agree	Neutral	Disagree	Strongly disagree
I find meaning in my role even if I do not succeed in helping a patient to gain insight into his or her illness	Strongly agree	Agree	Neutral	Disagree	Strongly disagree
I am pleased to help my friends/colleagues in their activities	Never/almost never	Rarely	Occasionally	Often	Always/almost always
I share the things that I have with my friends	Never/almost never	Rarely	Occasionally	Often	Always/almost always
I try to help others	Never/almost never	Rarely	Occasionally	Often	Always/almost always
I am available for volunteer activities to help those who are in need	Never/almost never	Rarely	Occasionally	Often	Always/almost always
I am empathic with those who are in need	Never/almost never	Rarely	Occasionally	Often	Always/almost always
I help immediately those who are in need	Never/almost never	Rarely	Occasionally	Often	Always/almost always
I do what I can to help others avoid getting into trouble	Never/almost never	Rarely	Occasionally	Often	Always/almost always
I intensely feel what others feel	Never/almost never	Rarely	Occasionally	Often	Always/almost always
I am willing to make my knowledge and abilities available to others	Never/almost never	Rarely	Occasionally	Often	Always/almost always
I try to console those who are sad	Never/almost never	Rarely	Occasionally	Often	Always/almost always
I easily lend money or other things	Never/almost never	Rarely	Occasionally	Often	Always/almost always
I easily put myself in the shoes of those who are in discomfort	Never/almost never	Rarely	Occasionally	Often	Always/almost always
I try to be close to and take care of those who are in need	Never/almost never	Rarely	Occasionally	Often	Always/almost always
I easily share with friends any good opportunity that comes to me	Never/almost never	Rarely	Occasionally	Often	Always/almost always
I spend time with those friends who feel lonely	Never/almost never	Rarely	Occasionally	Often	Always/almost always
I immediately sense my friends’ discomfort even when it is not directly communicated to me	Never/almost never	Rarely	Occasionally	Often	Always/almost always

Appendix B

The following is the consent form for participation in the study.

Title of the Study

Empathy, Moral Sensitivity, and Prosocialness Among Medical Undergraduates in a Tertiary Care Teaching Institute of South India - An Analytical Cross-Sectional Study

Principal Investigator

Sagnika Chowdhury Saveetha Medical College and Hospital, Saveetha Institute of Medical and Technical Sciences, Chennai, Tamil Nadu

Introduction

You are invited to participate in a research study that seeks to explore how different levels of empathy, moral sensitivity, and pro-socialness vary among medical students based on various factors affecting these attributes. Participation in this study is voluntary, and you can withdraw at any time without any consequences.

Purpose of the Study

This study aims to examine the levels of empathy, moral sensitivity, and pro-socialness among medical students and understand how these attributes are influenced by various factors such as academic year, personal experiences, and other demographics.

Procedures

If you agree to participate:

You will be asked to complete an online questionnaire about your empathy, moral sensitivity, and prosocialness.

The survey will take approximately 10-15 minutes.

No personal information will be collected to ensure your anonymity.

Risks and Discomforts

There are no known risks associated with this study. You can skip any questions that you feel uncomfortable answering.

Benefits

While there is no direct benefit to you, your participation will contribute to improving the understanding of how empathy, moral sensitivity, and prosocialness develop in medical students, which may influence future medical education strategies.

Confidentiality

Your responses will be kept anonymous, and no identifying information will be collected. Data will be stored securely and used for research purposes only. Your identity will remain confidential at all times.

Voluntary Participation

Participation is voluntary. You may withdraw from the study at any time without penalty or loss of benefits. If you choose to withdraw, your data will not be used in the analysis.

Questions and Concerns

If you have any questions or concerns regarding the study, you may contact the Principal Investigator at sagnika.chowdhury02@gmail.com.

Consent Statement

By selecting "Agree" below, you confirm that you have read and understood the information provided, and you voluntarily consent to participate in this study.

I agree to participate in this study.

I do not agree to participate in this study.

Thank you for your time and participation.
